# Bacterial Community Diversity and Screening of Growth-Affecting Bacteria From *Isochrysis galbana* Following Antibiotic Treatment

**DOI:** 10.3389/fmicb.2019.00994

**Published:** 2019-05-07

**Authors:** Jia-Yi Cao, Zhou-Yan Kong, Yu-Fan Zhang, Ting Ling, Ji-Lin Xu, Kai Liao, Cheng-Xu Zhou, Xiao-Jun Yan

**Affiliations:** ^1^Key Laboratory of Applied Marine Biotechnology, Ningbo University, Ministry of Education of China, Ningbo, China; ^2^Collaborative Innovation Center for Zhejiang Marine High-Efficiency and Healthy Aquaculture, Ningbo University, Ningbo, China

**Keywords:** *Isochrysis galbana*, antibiotics, bacterial community, growth-affecting bacteria, bacteria-algae interactions

## Abstract

Algal cultures are generally co-cultures of algae and bacteria, especially when considering outdoor cultivation. However, the effects of associated bacteria on algal growth remain largely unexplored, particularly in the context of *Isochrysis galbana*. In the present study, we investigated the effects of antibiotic on the growth of *I. galbana* and its associated bacterial community. We found advantageous responses of *I. galbana* to antibiotic exposure, evidenced by the increased growth, and the maximal photochemical efficiency of PSII (F_v_/F_m_). Since antibiotics can cause major disturbances within bacterial community, we further conducted 16S rDNA amplicon sequencing to determine the changes of bacterial community diversity following antibiotic treatment. We found that antibiotic treatment considerably and negatively affected the abundance and diversity of bacterial community, and 17 significantly decreased bacterial species in the antibiotic-treated medium, including *Pseudomonas stutzeri*, were identified. Further co-culture experiments revealed that *P. stutzeri* inhibited the growth of *I. galbana*, and the inhibitory activity was retained in the cell-free bacterial filtrate. These results indicated that the negative effect of bacteria was not exclusively transmitted through contact with *I. galbana* but could be also mediated via secretory compounds. Taken together, our findings not only fully characterized the bacterial community associated with *I. galbana* and how the bacterial community changed in response to antibiotic perturbations, but also provided a valuable information about the interactions between *I. galbana* and its associated bacteria, which might help improve the yield, and quality of *I. galbana* during its cultivation processes.

## Introduction

Microalgae with high efficiency of carbon dioxide fixation can produce and accumulate large amounts of macromolecules, such as carbohydrates, lipids and proteins, due to the rapid growth (Hemaiswarya et al., 2011). Some microalgae are essential food for marine grazers because of their proper nutritional value and digestibility (Gladue and Maxey, 1994). One of such microalgae is *Isochrysis galbana*, which contains high contents of long-chain polyunsaturated fatty acids (PUFAs), particularly docosahexaenoic acid (DHA), and eicosapentaenoic acid (EPA) (Sanchez et al., 2000). This microalgal strain has been widely used as feed for bivalves, fish, zooplankton as well as crustaceans (Su et al., 2017). Especially, *I. galbana* has been considered as the essential diet of many bivalve mollusks when they are just hatched during artificial rearing. A great deal of attention has been paid to *I. galbana* due to its important nutritional value and potential application in aquaculture.

For a wide application of *I. galbana* in aquaculture, the key point is to find strategies to improve outdoor culture systems. Tremendous efforts have been made to study the effects of environmental factors on *I. galbana*, such as temperature, light condition, photoperiod, and trophic conditions (Babuskin et al., 2014; Alkhamis and Qin, 2015; Nogueira et al., 2015; Gim et al., 2016; Su et al., 2017). Previous investigations have well explored the effects of different carbon source combinations on biomass and lipid production of *I. galbana* under different photoperiods and light intensities (Babuskin et al., 2014). It has been reported that combined effects of temperature (30°C) and light intensity (400 mmol photons m^-2^ s^-1^) significantly enhance the TAG content and productivity of *I. galbana* (Nogueira et al., 2015). The changes of superoxide dismutase activity, organic acid content, and lipid level at different growth stages at 35°C indicate that high temperature has negative effects on the growth and metabolism of *I. galbana* (Su et al., 2017). Taken together, environmental factors play vital roles in the growth and metabolism of *I. galbana*.

In addition to these above-mentioned environmental factors (also considered as abiotic factors), increasing evidence has suggested that biotic factors, especially algae-associated bacteria, have significant effects on the physiology, and metabolism of algae by certain complex interactions and communication mechanisms (Natrah et al., 2014; Amin et al., 2015; Cooper and Smith, 2015; Ramanan et al., 2016; Mayali, 2018). These types of algae-bacteria interactions have been shown to facilitate the growth of algae and protect them from invading pathogens, or to inhibit the growth of algae. Therefore, knowledge of the bacterial community associated with algae is of great importance for understanding homeostasis in robust algal cultures. It is interesting to find that a *Sulfitobacter* species can promote *Pseudonitzschia multiseries* cell division via secretion of the hormone indole-3-acetic acid (Amin et al., 2015). *Ruegeria pomeroyi* DSS-3 exchanges organosulfur compounds with *Thalassiosira pseudonana*, which alleviates the vitamin B_12_ limitation of the diatom (Durham et al., 2015). The interaction between them is found to be regulated by a range of genes involved in response to external stimuli, lipid and chitin biosynthesis in the diatom (Durham et al., 2017). More interestingly, bacteria play an immediate effect on breaking down organic phosphorus in order to provide nutrients for dinoflagellates (Cui et al., 2016; Wang et al., 2017). Apart from positive effects on algal growth, negative effects of bacteria on microalgal cultures have also been widely reported (Pei et al., 2007; Lakaniemi et al., 2012; Paul and Pohnert, 2013; Unnithan et al., 2014; Fulbright et al., 2016). For example, *Bacillus pumilus*, isolated from a poorly performing *Nannochloropsis salina* culture, is found to inhibit the growth of *N. salina*. Furthermore, the inhibitory effect of *B. pumilus* culture filtrate on *Nannochloropsis* sp. suggests that an active molecule is released into the culture (Fulbright et al., 2016). It has been determined that *Kordia algicida* can lyse *Phaeodactylum tricornutum, T. weissflogii*, and *Skeletonema costatum* by releasing a special protease (Paul and Pohnert, 2013). Collectively, the interaction between algae and their associated bacteria has a non-negligible effect on the physiology and metabolism of algae. Therefore, optimal cultivation conditions of *I. galbana* should always take its associated bacterial community into account. However, limited information is available on the bacterial community associated with *I. galbana*, which significantly hinders us to understand its effects on the long-term stability of *I. galbana* culture.

In the present study, we mainly aimed to characterize how *I. galbana* and its associated bacterial community changed in response to antibiotic perturbations, as well as the effect of bacteria on the growth of *I. galbana*. We firstly evaluated the effects of antibiotic on the growth of *I. galbana*. Moreover, the changes of bacterial community diversity following antibiotic treatment were analyzed by 16S rDNA amplicon sequencing. Furthermore, we had found some growth-affecting bacteria from *I. galbana* based on the analysis of sequencing results. Our findings not only promoted a better understanding of bacteria-*I. galbana* interactions, but also provided an important reference for its cultivation.

## Materials and Methods

### Culture of Microalgae

*Isochrysis galbana* was obtained from the Marine Biotechnology Laboratory of Ningbo University, China. As culture medium, the seawater was filtered through 0.45-μm cellulose acetate membranes and then sterilized by autoclaving. NMB3 medium used in this study was composed of KNO_3_ (100 mg/L), KH_2_PO_4_ (10 mg/L), MnSO_4_^⋅^H_2_O (2.5 mg/L), FeSO_4_^⋅^7H_2_O (2.5 mg/L), EDTA-Na_2_ (10 mg/L), vitamin B1 (6 μg/L), and vitamin B12 (0.05 μg/L) (Yang et al., 2016). All the microalgae were cultivated at the light intensity of 100 μmol photon m^-2^ s^-1^.

### Antibiotic Treatment

*Isochrysis galbana* in the early exponential growth phase was cultured in NMB3 medium supplemented with ampicillin (500 μg/mL), which was denoted as the antibiotic-treated (AT) group. Such antibiotic treatment lasted for 6 days. The cultures were carefully shaken by hand once daily to ensure a good homogenization of antibiotic and microalgae. In parallel, *I. galbana* was cultured in triplicate under the above-mentioned conditions without antibiotic, which was denoted as the CK group. Samples were collected every day for microalgal cell counting and chlorophyll fluorescence measurement until the end of the experiment.

### Measurements of the Growth and Chlorophyll Fluorescence

The microalgal cell density was daily monitored using a microscope and Hausser hemocytometer until the end of the experiment. Specific growth rate (μ) for each day was calculated according to the following equation: μ = lnCt_2_–lnCt_1_, where Ct_2_ and Ct_1_ are cell density at time point t_2_ (day) and the day before t_2_, respectively. To analyze the photosynthetic response of antibiotic-treated algal cells, the maximum quantum yield of PS II (F_v_/F_m_), and the non-photochemical quenching (NPQ) were investigated using fast chlorophyll fluorescence with a Water-PAM (Heinz Walz, Germany) as previously described (Zhang R. et al., 2017).

### Determination of the Bacterial Community in *I. galbana* Culture

#### DNA Extraction and PCR Amplification

Bacterial genomic DNA was extracted from *I. galbana* cultures in quadruplicate at the end of the growth experiment (day 6) as previously described (Xiong et al., 2014). DNA concentration and purity were examined by electrophoresis on 1% agarose gels, and the final concentration of purified DNA was adjusted to 1 ng/μL using sterile water.

Before sequencing, the extracted DNA was amplified using specific primers for hypervariable V4 region of the 16S rDNA. The forward and reverse primers were 515F (5′-GTGCCAGCMGCCGCGGTAA-3′) and 806R (5′-GGACTACHVGGGTWTCTAAT-3′), respectively (Caporaso et al., 2011). PCR was performed using Phusion^®^ High-Fidelity PCR Master Mix (New England Biolabs, United States). The PCR products were detected by electrophoresis on 2% agarose gels, and equal amounts of amplicons and purified with Qiagen Gel Extraction Kit (Qiagen, Germany).

#### Library Preparation and High-Throughput Sequencing Analysis

Libraries were prepared by TruSeq^®^ DNA PCR-Free Sample Preparation Kit (Illumina, United States) following the manufacturer’s instructions. The Qubit@ 2.0 Fluorometer (Thermo Fisher Scientific) and Agilent Bioanalyzer 2100 system were applied to assess the quality of libraries. Finally, the libraries were sequenced on an Illumina HiSeq 2500 platform, and 250-bp paired-end reads were generated (Novogene Co., Ltd., Beijing, China).

Sequences were analyzed by both QIIME (version 1.9.1) and the UPARSE pipeline (Caporaso et al., 2010). The default settings used for these two types of software were then applied to assign taxonomy at an identity of 97% with the RDP Classifier (Wang et al., 2007) based on SILVA database (Quast et al., 2013). After taxa were assigned, operational taxonomic units (OTUs) affiliated with Archaea, Chloroplasts, and unclassified (not affiliated with bacteria) were removed from the dataset. To avoid artifacts resulting from sample size, a randomly selected subset of 62,098 sequences (corresponding to the smallest sequencing effort for any of the samples) per sample was used for downstream analysis. Alpha diversity indices (Observed-species, Shannon, Simpson, Chao1, ACE, Good-coverage, and PD whole tree) were calculated based on OTUs and the phylogenetic tree using the alpha_diversity.py in the QIIME pipeline. The raw data generated in this study were deposited in the DDBJ^1^ Sequence Read Archive under the accession number of DRA007640.

### Statistical Analysis

All statistical analyses were performed in the R-environment^2^ unless otherwise indicated (Yang et al., 2018). Cluster analysis was conducted by principal component analysis (PCA), which was applied to reduce the dimension of the original variables using the FactoMineR package and ggplot2 package in R software (Version 3.5.1). Permutational multivariate analysis of variance (PERMANOVA) with the function “adonis” in the “vegan,” analysis of similarity (Anosim) with the function “anosim,” multi response permutation procedure (MRPP) with the function mrpp, and analysis of molecular variance (Amova) with the function amova were performed to test significant differences between different treatments. Significantly changed bacteria at different taxonomic levels were examined using MetaStat method (White et al., 2009). *I. galbana* growth indexes were analyzed using SPSS 22.0 for Windows, and Student’s *t*-test was performed to determine the significant difference between two samples. A *p* value < 0.05 was considered as statistically significant. Data in the paper were given as mean ± SE.

### Generation of Axenic *I. galbana* Cultures

Axenic *I. galbana* cultures were generated using a previously described method with minor modifications (Amin et al., 2015). Approximately 50 mL of *I. galbana* culture at mid-exponential phase was centrifuged at 5,000 rpm for 10 min, and the pellet was washed with fresh NMB3 medium for two times and resuspended in the same medium. Subsequently, *I. galbana* culture was filtered using 5-μm polycarbonate membrane filter (Millipore). Cells were quickly rinsed with sterile seawater. The remaining steps to generate axenic *I. galbana* cultures were the same as the previously described protocol except for the concentration of antibiotics. The antibiotics used in the present study included streptomycin (100 μg/mL), gentamycin (134 μg/mL), ciprofloxacin (40 μg/mL), chloramphenicol (4.4 μg/mL), and ampicillin (200 μg/mL). The presence of bacteria was assessed after subculturing in antibiotic-free medium for three times in order to remove the antibiotics. Three methods were used to detect the presence of bacteria in *I. galbana* cultures. First, the treated algal cultures were monitored for bacterial contamination by checking for bacterial growth on 2216E agar plates, and untreated cultures were employed as controls. No bacteria were detected on the agar plates after incubation for 7 days (Supplementary Figure S1A). Second, community DNA of treated and untreated algal cultures was extracted as described previously after filtration of *I. galbana* cells (Xiong et al., 2014). The 16S rDNA of bacteria was amplified by PCR using 27F and 1492R primers and the extracted DNA as template. Bacterial 16S rDNA was detected in the untreated cultures but not detected in the antibiotic-treated cultures (Supplementary Figure S1B). Third, both treated and untreated algal cultures (with *I. galbana* cells) were used to isolate total DNA for PCR amplification, and the PCR products were then cloned into *E. coli* vector. For antibiotic-treated cultures, analysis of 30 recombinant clone sequences revealed that all the inserts were highly similar with organelle 16S rRNA genes of *I. galbana* and other microalgae, while none of them were bacterial-specific. All these results showed that bacteria were absent in the antibiotic-treated algal cultures.

### Co-culture Experiments

Briefly, 2216E agar plates were used to isolate bacteria from *I. galbana* culture. The 16S rRNA genes of the isolated bacteria were amplified by PCR using universal bacterial primers 27F and 1492R. The amplified products were gel purified and ligated into pMD19-T vector for sequencing. The almost-complete 16S rRNA gene sequence was compared with reference sequences in NCBI by BLAST^3^. Based on the result of 16S rRNA gene sequence alignment and phylogenetic analysis, one isolated bacterial strain (namely IG-3) shared 100% sequence identity to the validly named species *Pseudomonas stutzeri*. For co-culture experiments, single colony of this isolated *P. stutzeri* was freshly plated before each experiment on 2216E agar plates. *P. stutzeri* was inoculated into 2216E broth and incubated for 24 h in a shaking incubator (25°C, 180 rpm). Freshly prepared bacterial cells (OD_600_ = 0.4–0.6) were centrifuged (5,000*g* for 5 min) and washed twice with sterile NMB3 medium. When axenic *I. galbana* was cultured to exponential phase (cell density of about 1 × 10^6^ cells/mL), *P. stutzeri* was added into algal culture to achieve a bacteria/algae ratio of about 20:1 (cell counts: cell counts). During the course of 6-day co-culture, the algal growth was determined daily by monitoring levels of chlorophyll as previously described (Bruckner et al., 2008), and the bacterial growth was measured by counting colony-forming units.

For generation of cell-free bacterial filtrate, *P. stutzeri* was inoculated into 50 mL of 2216E broth and grown to stationary phase at 28°C for 36 h on a shaker at 150 rpm. The cell-free supernatant was collected by centrifugation at 14,000 rpm for 8 min and then gently filtered through a 0.22-μm Millipore membrane. The filtrate was added to the algal cultures at a concentration of 5% or 10% (vol/vol) to assess their effects on the growth of *I. galbana*. In parallel, an algal culture supplemented with the same volume of sterile 2216E medium served as the control group.

## Results

### The Effects of Antibiotic on the Growth and Photosynthetic Properties of *I. galbana*

**FIGURE 1:**
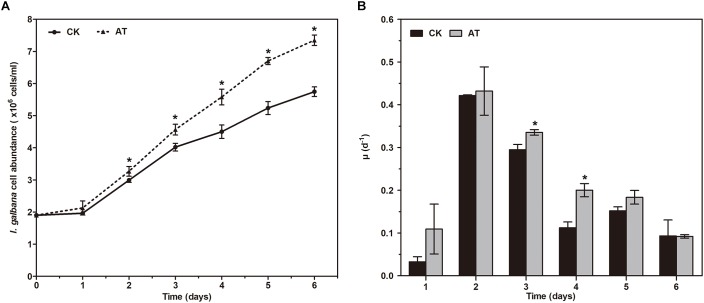
Growth of *Isochrysis galbana* in the antibiotic-treated and untreated culture expressed as cell concentration **(A)** and growth rate **(B)**. Significance of the differences between mean values was determined with Student’s *t* test. Error bars represent SE, while asterisks (^∗^) indicate significant difference at *p* < 0.05.

The growth of *I. galbana* in the antibiotic-treated (denoted as AT) or untreated (denoted as CK) medium was compared. The addition of antibiotic had a significant effect on the growth of *I. galbana* (Figure 1A). During 6 days of the experiments, *I. galbana* cultivated in the AT medium exhibited higher cell density compared with the CK medium (Figure 1A). In addition, as culture time was increased, the difference in cell abundance of *I. galbana* cultured under these two conditions became greater. Besides, the growth rate in the AT group on the 3rd and 4th day was significantly higher than that in the CK group (Figure 1B). All these data clearly showed that *I. galbana* cultured in the antibiotic-treated medium grew faster compared with the untreated *I. galbana*.

**FIGURE 2:**
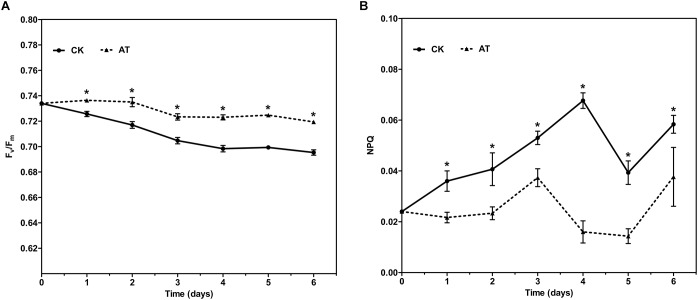
F_v_/F_m_ **(A)** and NPQ **(B)** for control and antibiotic-treated cultures of *I. galbana* during 6 days exposure time. Significance of the differences between mean values was determined with Student’s *t* test. Error bars represent SE, while asterisks (^∗^) indicate significant difference at *p* < 0.05.

The effects of antibiotic treatment on the photosynthetic property of *I. galbana* were evaluated by monitoring the chlorophyll fluorescence parameters every day. The maximal photochemical efficiency of PSII (F_v_/F_m_) was increased with the addition of antibiotic in the whole culture process compared with the controls, while NPQ was only slightly decreased (Figure 2). The results revealed that photosynthetic property of *I. galbana* were improved when the antibiotic was added into the culture medium.

### Responses of Bacterial Community Associated With *I. galbana* to Antibiotic Treatment

Since *I. galbana* grew better in the antibiotic-treated medium compared with the antibiotic-free medium, and it is well known that antibiotics can cause significant disturbance of microbiomes, we hypothesized that the bacteria with significantly changed abundance upon antibiotic treatment might have effect on the growth of *I. galbana*. Therefore, 16S rDNA amplicon sequencing was conducted to determine the bacterial community associated with antibiotic-treated (AT) and untreated (CK) *I. galbana* cultures. Four different cultures for each treatment and denoted as AT and CK group, respectively. By HiSeq sequencing analysis, 62,591–93,213 effective sequence tags were obtained for each of these 8 samples (Supplementary Table S1). The read length of nearly all the reads ranged from 200 to 300 bp, and the average length was 253 bp. Q20 and Q30 were both higher than 98%, while the effective% was all higher than 93.75%. All these data indicated that the quality of the sequencing results met the requirements for subsequent analysis.

**FIGURE 3:**
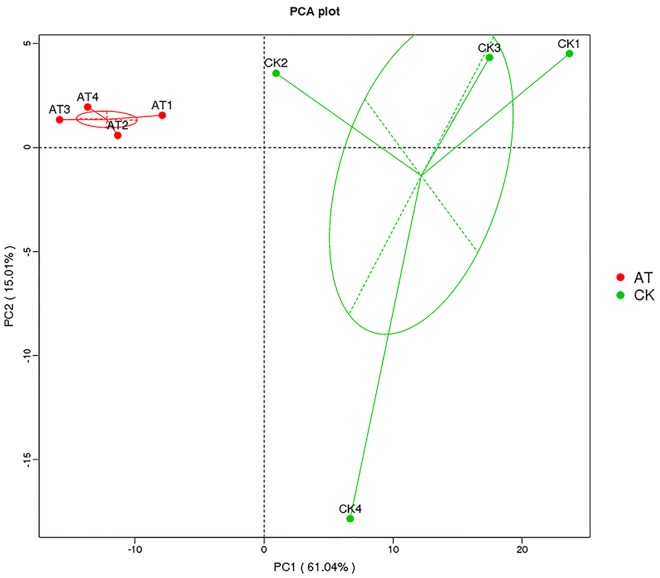
Principal component analysis (PCA) of the associated bacterial community of *I. galbana* from AT and CK groups. In each sample name, AT, or CK represents the *I. galbana* samples from antibiotic-treated or untreated culture, respectively; the arabic number (1, 2, 3, or 4) after AT or CK represents the four replicate samples.

The average number of OTUs observed in the AT group was 553, which was significantly lower than that in the CK group (1,189) (Table 1). All seven alpha diversity indices from the AT group were also significantly lower than those in the CK group (*p* < 0.05) (Table 1). Rarefaction curves further confirmed that the microbiome of the AT group was less diverse compared with the CK group (Supplementary Figure S2). All these results suggested that bacteria associated with *I. galbana* grown in antibiotic-treated medium were less diverse than those in the CK group. Furthermore, all the four samples from the antibiotic-treated and control groups were distributed in different parts of PCA plot (Figure 3), indicating that the bacterial community of AT and CK groups was different from each other. Besides, four nonparametric multivariate statistical tests, including Adonis, Anosim, MRPP and Amova, were performed to determine whether the sample groups (different treatments) had significant differences. Table 2 shows that the values of R_Anosim_ and A_MRPP_ were both greater than zero, and *p* values obtained from these four analyses were all less than 0.05. All these results indicated that the difference between groups was greater than that within groups, and such difference was significant (*p* < 0.05). Therefore, Adonis, Anosim, MRPP, and Amova confirmed the presence of distinctly different bacterial community associated with *I. galbana* in the antibiotic-treated and control cultures (Table 2).

**Table 1:** Comparsion of bacterial community α-diversity indices (mean ± SE) between antibiotic-treated and control samples.

Sample name	OTU	Observed-species	Shannon	Simpson	Chao1	ACE	Goods-coverage	PD whole tree
CK	1189	974 ± 83.36^a^	3.16 ± 0.33^a^	0.59 ± 0.07^a^	1142.42 ± 88.89^a^	1234.90 ± 91.15^a^	0.995 ± 0^a^	77.62 ± 4.71^a^
AT	553	432 ± 83.53^b^	1.18 ± 0.13^b^	0.24 ± 0.02^b^	660.17 ± 73.55^b^	745.33 ± 85.37^b^	0.997 ± 0^b^	41.97 ± 7.07^b^


*^*ab*^The various letters indicate significant differences between two groups.*

**Table 2:** Significance tests of the effects of antibiotic on the overall bacterial community structure with four different statistical approaches.

	Adonis		Anosim		MRPP		Amova	
				
	*R*^2^	*P*-value	*R*-value	*P*-value	A	*P*-value	Fs	*P*-value
16S rRNA gene 97% cutoff	0.528	0.001	0.583	0.032	0.331	0.023	10.633	0.022


### Bacterial Community Composition Modulation Upon Antibiotic Treatment

**FIGURE 4:**
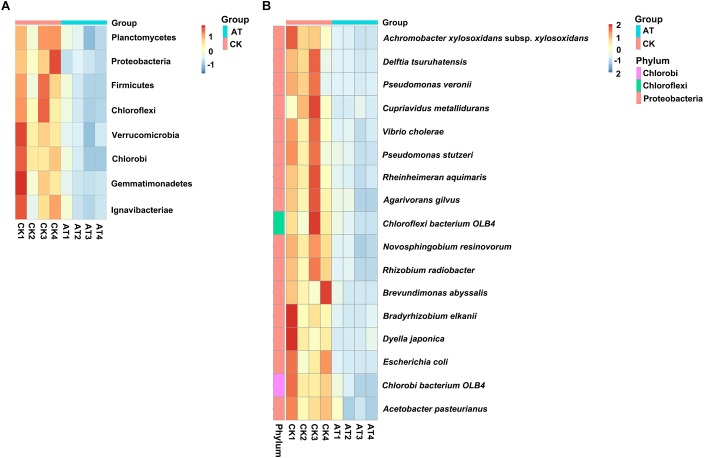
Heat map showed the relative abundance of the significantly changed bacteria at phylum **(A)** and species **(B)** taxonomic level based on MetaStat method. Origin of microbiota is indicated either as AT (green) or CK (red) samples. Relative abundance from high to low of bacteria was indicated with color from red to blue.

To understand which specific bacterial populations might be affected by antibiotic, the significantly changed bacteria at different taxonomic levels were identified by MetaStat method (Supplementary Table S2). At the 97% OTU level, 123 significantly changed phylotypes were found, and all of them were decreased in response to antiobiotic treatment (Supplementary Figure S3). At the phylum level, the relative abundance of bacteria affiliated with *Planctomycetes, Proteobacteria, Firmicutes, Chloroflexi, Verrucomicrobia, Chlorobi, Gemmatimonadetes*, and *Ignavibacteriae* was significantly decreased (*p* < 0.05) (Figure 4A). Further analysis of those significantly changed phyla revealed that those changes occurred in some specific bacterial groups at the class or lower taxonomic levels (Supplementary Table S2). For example, in *Planctomycetes*, all significant changes (*p* < 0.05) occurred in *Brocadiales* and *Phycisphaerales* order, which were affiliated with *Planctomycetacia* and *Phycisphaerae* class, respectively. To further examine effects of antibiotic on bacterial community composition at the species level, we found that the relative abundance of 17 species was significantly (*p* < 0.05) decreased in the antibiotic-treated group (Figure 4B). Except for two species, all the other 15 significantly changed species were affiliated with *Proteobacteria*. Therefore, all these results indicated that the overall taxonomic composition and structure of bacterial community were significantly affected by antibiotic treatment.

### *Pseudomonas stutzeri* Inhibits the Growth of *I. galbana*

**FIGURE 5:**
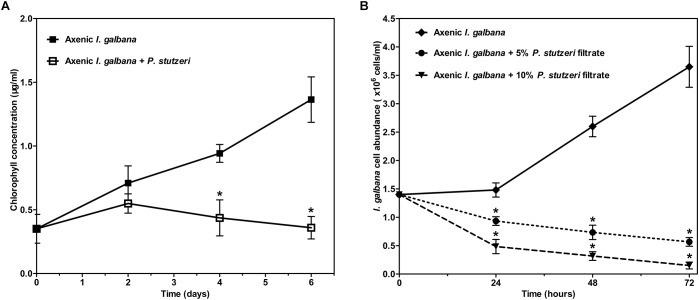
The negative effect of *P. stutzeri* cells **(A)** and cell-free bacterial filtrate **(B)** on the growth of *I. galbana*. Values are means standard errors (SEs) (*n* = 3), while asterisks (^∗^) indicate significant difference at *p* < 0.05.

All the above-mentioned results could be concluded briefly. The relative abundance of 17 species was significantly decreased in the antibiotic-treated group compared with the control group. Meanwhile, *I. galbana* grew better in the antibiotic-treated medium. Therefore, we hypothesized that these bacteria inhibited by antibiotic might have effects on the growth of *I. galbana*. We tested the effects of one of these 17 species (*P. stutzeri*), isolated from *I. galbana* culture, on the growth of *I. galbana* by co-culture experiments. Co-culture of *I. galbana* and *P. stutzeri* decreased the chlorophyll contents by 23–74% compared with the axenic culture over the period of 6 days, suggesting that cultures with bacteria grew slower than the axenic *I. galbana* culture (Figure 5A). To further explore the mechanism underlying the inhibitory effect, we assessed the effects of cell-free filtrate of *P. stutzeri* at different concentrations (5 and 10%) on the growth of *I. galbana*. Similar with the results of co-culture of *I. galbana* and *P. stutzeri*, the filtrate of active *P. stutzeri* cultures significantly decreased the cell growth of *I. galbana* compared with the control group (Figure 5B). Besides, the algicidal effects were enhanced with the increase of concentration and treatment duration (Figure 5B). Taken together, *P. stutzeri* could inhibit the growth of *I. galbana* in a direct contact manner (Figure 5A). In addition, the negative effects of the bacteria could be retained in the cell-free filtrate of *P. stutzeri* (Figure 5B).

## Discussion

*Isochrysis galbana* has been widely used in aquaculture as feed for fish larvae, mollusks and crustaceans due to its high nutritional value (Sanchez et al., 2000; Yang et al., 2016). However, there is still a big challenge in the outdoor mass cultivation of *I. galbana*, which limits wide application of this microalgal strain in aquaculture. In our current study, we uniquely aimed to investigate the effects of the associated bacterial community on the growth of *I. galbana*, which might play vital roles in regulating the growth of this microalgal strain. Our results indicated that antibiotic-treatment significantly affected the growth of *I. galbana* and its associated bacterial community. Antibiotic altered the *I. galbana*-associated bacterial community diversity, which might be attributed to its growth-promoting effect on *I. galbana*. Therefore, our results provided new insights into the cultivation of *I. galbana*. Furthermore, this was our first attempt to study bacterial community associated with *I. galbana* and its effect on the growth of *I. galbana* based on high-throughput sequencing.

Interestingly, we identified the advantageous response of *I. galbana* to antibiotic exposure, evidenced by the increased growth rate and F_v_/F_m_. First of all, antibiotic-treatment clearly improved the growth of *I. galbana*, and the difference between AT and CK groups became greater on the 3rd and 4th day (Figure 1). These results could be explained by that the antibiotic exerted its effect on bacterial community in the culture in a time-dependent manner. Besides, F_v_/F_m_, a photosynthesis parameter reflecting the maximum quantum yield of PSII when all reaction centers are open (Schreiber et al., 1995), was significantly increased in *I. galbana* grown in the antibiotic-treated medium. NPQ, another chlorophyll fluorescence parameter, reflects the ability of plant to dissipate energy, which is directly related to the ability to provide photoprotection to plant (Bachmann et al., 2004). Previous studies have documented that photosynthetic organisms would elevate NPQ when exposed to some environmental stressors. For example, it has been reported that high light intensity stress induces a decrease of F_v_/F_m_ and an increase of NPQ of *T. pseudonana* (Zhang R. et al., 2017). In addition, *Karlodinium veneficum* cells respond to P deprivation by reconfiguring the metabolic landscape and up-tuning NPQ to increase the capacity to dissipate excess light energy and maintain the fluency of energy flow (Cui et al., 2017). In the present study, the values of NPQ in both AT and CK groups were low, while NPQ in the AT group was even lower than that of the CK group (Figure 2). These findings suggested that the antibiotic treatment could improve the photosynthetic function of *I. galbana*. Moreover, the antibiotic treatment promoted the growth of *I. galbana*, evidenced by all these above-mentioned results. It was noteworthy that the antibiotic used here was ampicillin and its concentration was 500 μg/mL. However, it has been reported that some antibiotics, such as chloramphenicol, florfenicol, thiamphenicol, erythromycin and furazolidone, can inhibit the growth of *I. galbana* (Campa-Cordova et al., 2006; Lai et al., 2009). Surprisingly, we found that ampicillin at a concentration of 1,000 μg/mL inhibited the growth of *I. galbana* (Supplementary Figure S4). This result might be attributed to that higher concentration of ampicillin had a direct adverse effect on the growth of *I. galbana* like those above-mentioned antibiotics. Taken together, the type and concentration of antibiotics were both important factors. Therefore, we could speculate that ampicillin exerted its promoting effect on the growth of *I. galbana* indirectly.

Algal cultures are usually found to be co-cultures of algae and bacteria, especially when considering outdoor cultivation. It is known that a complex bacterial community associated with algae can affect many aspects of algal host, such as the growth, behavior, and physiology (Natrah et al., 2014; Amin et al., 2015; Cooper and Smith, 2015; Ramanan et al., 2016; Mayali, 2018). Moreover, antibiotics can significantly disturb the ecological balance of microbiome (Mayali and Azam, 2004), and antibiotics are always used to control the bacterial population in aquaculture. Furthermore, since there are beneficial bacteria associated with algae, it is inappropriate to indiscriminately kill all bacteria using non-targeted antibiotics. In the present study, the addition of ampicillin promoted the growth of *I. galbana*, which might be attributed to that the growth-promoting bacteria were induced or the harmful bacteria were inhibited. To substantiate this hypothesis, we assessed the bacterial community modulation of *I. galbana* culture in the presence or absence of antibiotic based on the diversity of the 16S rRNA genes by high-throughput sequencing technologies. Not surprisingly, there was an overall reduction in the OTU number in the AT group compared with the CK group, indicating that this antibiotic as a whole was effective to inhibit part of the bacteria associated with *I. galbana*. Besides, beta diversity analysis indicated that the bacterial community structure of AT and CK groups was significantly different from each other (Figure 3 and Table 2). More importantly, the relative abundance of 17 species was significantly (*p* < 0.05) decreased in the AT group (Figure 4B). Except for two species, all the others 15 species were affiliated with *Proteobacteria*, one of the most ubiquitous and abundant bacteria associated with algae. A number of publications have found that bacteria affiliated with *Proteobacteria* affect the growth of microalgae, including *Phaeodactylum tricornutum* and *T. pseudonana* (Zecher et al., 2015). Considering the results obtained in the current study, it could be speculated that bacteria affiliated with the phylum *Proteobacteria* were very important parts of microbial community associated with microalgae. It was worth mentioning that among these 17 significantly decreased species, two bacterial strains were affiliated with *Pseudomonas* genus, including *P. veronii*, and *P. stutzeri*. It has been reported that *Pseudomonas* sp. inhibits the growth of two marine algal strains, *Heterosigma akashiwo* and *Tetraselmis indica* (Wang et al., 2005; Arora et al., 2012). Therefore, we could speculate that *P. stutzeri* was also detrimental to *I. galbana* based on the results of co-culture experiments (Figure 5). *P. stutzeri*, isolated from *I. galbana* culture, had a negative effect on the growth of *I. galbana*. In a direct contact situation, a significantly inhibited growth of *I. galbana* was observed during the whole co-culture experiment. The negative effect of the bacteria was not exclusively transmitted through contact with *I. galbana* but could be also mediated via secretory compounds. This was clearly demonstrated by the fact that the activity of *P. stutzeri* medium remained after removal of the cells by sterile filtration. Relative inhibition of growth could be observed within the first day of incubation, indicating rapid action of the algicidal compounds. However, what exactly are the algicidal compounds and what are the algicidal mechanisms merit further investigations. Previous findings have shown that bacteria inhibit the growth of algae by secreting algicidal compounds (Zhang et al., 2016, 2018; Zhang H. et al., 2017; Wu et al., 2017). Taken together, we proposed that these significantly decreased bacteria, if not all, might wield effects on the growth of *I. galbana*. However, we have to isolate the remaining 16 species from *I. galbana* for co-culture experiments, and further studies are required in order to elucidate the effects of these species on *I. galbana*, as well as the bacteria-algae interactive mechanisms.

## Conclusion

Previous studies have barely investigated the bacterial community associated with *I. galbana* and its effects on the growth of this alga, despite the importance of bacterial community in phytoplankton ecology. Our current study, for the first time, extensively analyzed the bacterial communities associated with *I. galbana* by 16S rDNA amplicon sequencing. We found the advantageous responses of *I. galbana* to antibiotic exposure, which were mainly evidenced by the increased growth and F_v_/F_m_. Based on further 16S rDNA amplicon sequencing analysis, we speculated that the antibiotic-induced growth of *I. galbana* might be explained by that ampicillin altered the *I. galbana*-associated bacterial community diversity. Co-culture experiments revealed that *P. stutzeri*, one of 17 significantly decreased bacterial species in the antibiotic-treated group, inhibited the growth of *I. galbana*. The inhibitory activity was retained in the cell-free bacterial filtrate, demonstrating that the negative effect of the bacteria was not exclusively transmitted through contact with *I. galbana* but could be also mediated via secretory compounds. Collectively, bacterial community could have vital influence on the growth of *I. galbana*, and knowledge should be taken into consideration in the mass culture of this microalgal strain for its potential application in aquaculture.

## Author Contributions

J-LX coordinated the project. Z-YK, Y-FZ, and TL conducted the experiments. J-YC designed and performed the bioinformatics and statistical analysis. C-XZ and KL helped in analyzing and modeling the data. J-LX and X-JY conceived of the study and participated in its design and coordination. J-LX and J-YC prepared and revised the manuscript. All authors had read and approved the final manuscript.

## Conflict of Interest Statement

The authors declare that the research was conducted in the absence of any commercial or financial relationships that could be construed as a potential conflict of interest.

## Footnotes

1 http://www.ddbj.nig.ac.jp/

2 http://www.r-project.org

3 https://blast.ncbi.nlm.nih.gov/Blast.cgi
