# Supplementary material for: Bacterial Community Diversity and Screening of Growth-Affecting Bacteria From Isochrysis galbana Following Antibiotic Treatment
Source: Front Microbiol. 2019 May 7;10:994. doi: 10.3389/fmicb.2019.00994 (PMC6513876; doi:10.3389/fmicb.2019.00994)
Supplement: Supplementary file 3 [file Data_Sheet_1.PDF]

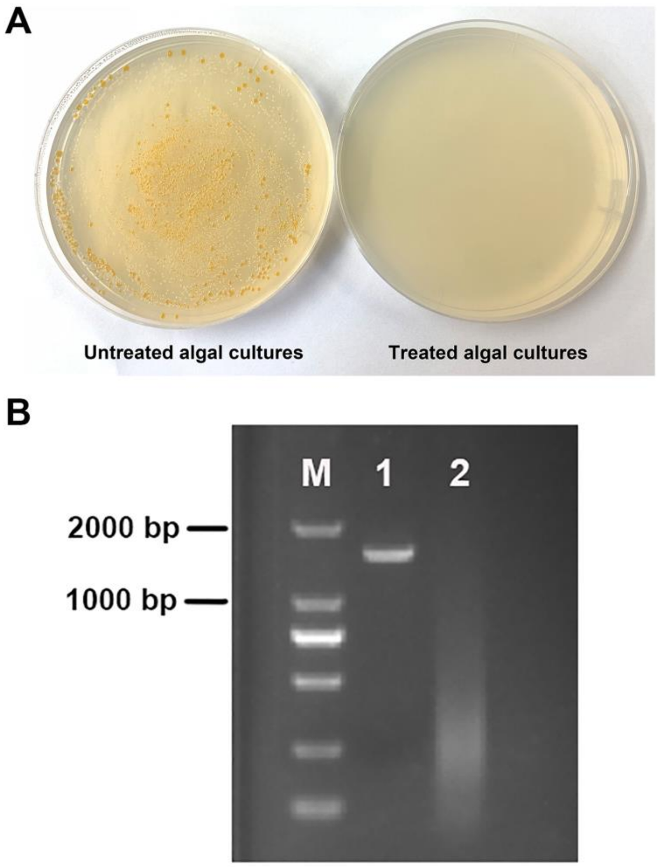

Figure S1. Two methods used to detect the presence of bacteria in *I. galbana* cultures. (A) 2216E agar plates for monitoring bacterial contamination. The untreated and treated algal cultures, all in triplicate, were monitored for bacterial contamination by plating on 2216E agar plates. Pictures were taken after incubation for 7 days. (B) Results of PCR amplification of 16S rDNA obtained from the untreated culture (Lane 1) and treated culture (Lane 2) DNA.
