# Supplementary material for: Bacterial Community Diversity and Screening of Growth-Affecting Bacteria From Isochrysis galbana Following Antibiotic Treatment
Source: Front Microbiol. 2019 May 7;10:994. doi: 10.3389/fmicb.2019.00994 (PMC6513876; doi:10.3389/fmicb.2019.00994)
Supplement: Supplementary file 4 [file Data_Sheet_2.PDF]

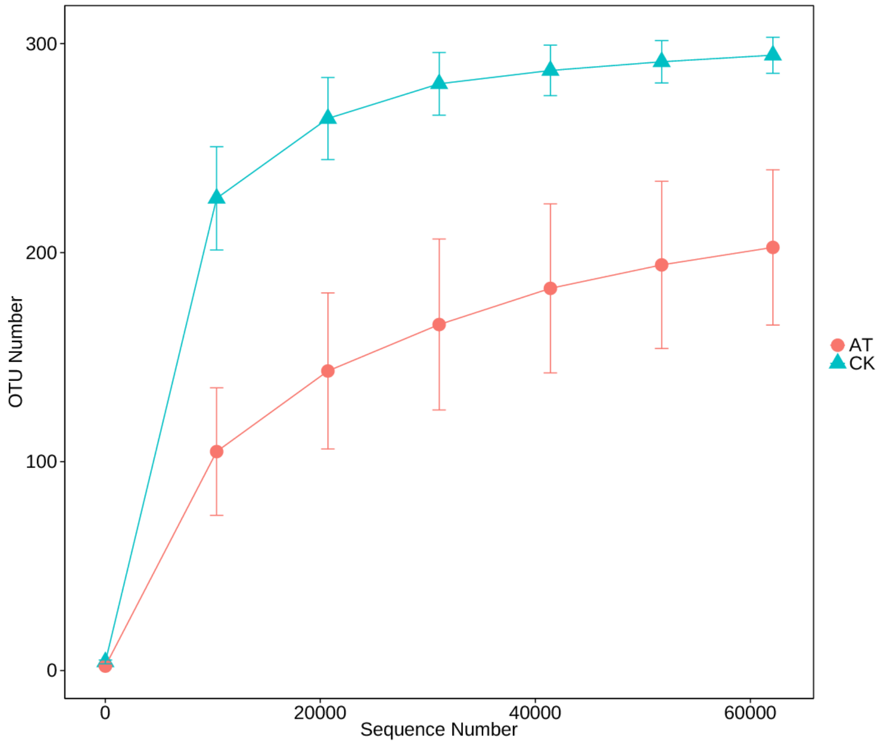

Figure S2. Rarefaction curves for antibiotic-treated (AT) and the control (CK) groups (based on OTUs at 97% identity).
