# Supplementary material for: Bacterial Community Diversity and Screening of Growth-Affecting Bacteria From Isochrysis galbana Following Antibiotic Treatment
Source: Front Microbiol. 2019 May 7;10:994. doi: 10.3389/fmicb.2019.00994 (PMC6513876; doi:10.3389/fmicb.2019.00994)
Supplement: Supplementary file 6 [file Data_Sheet_4.PDF]

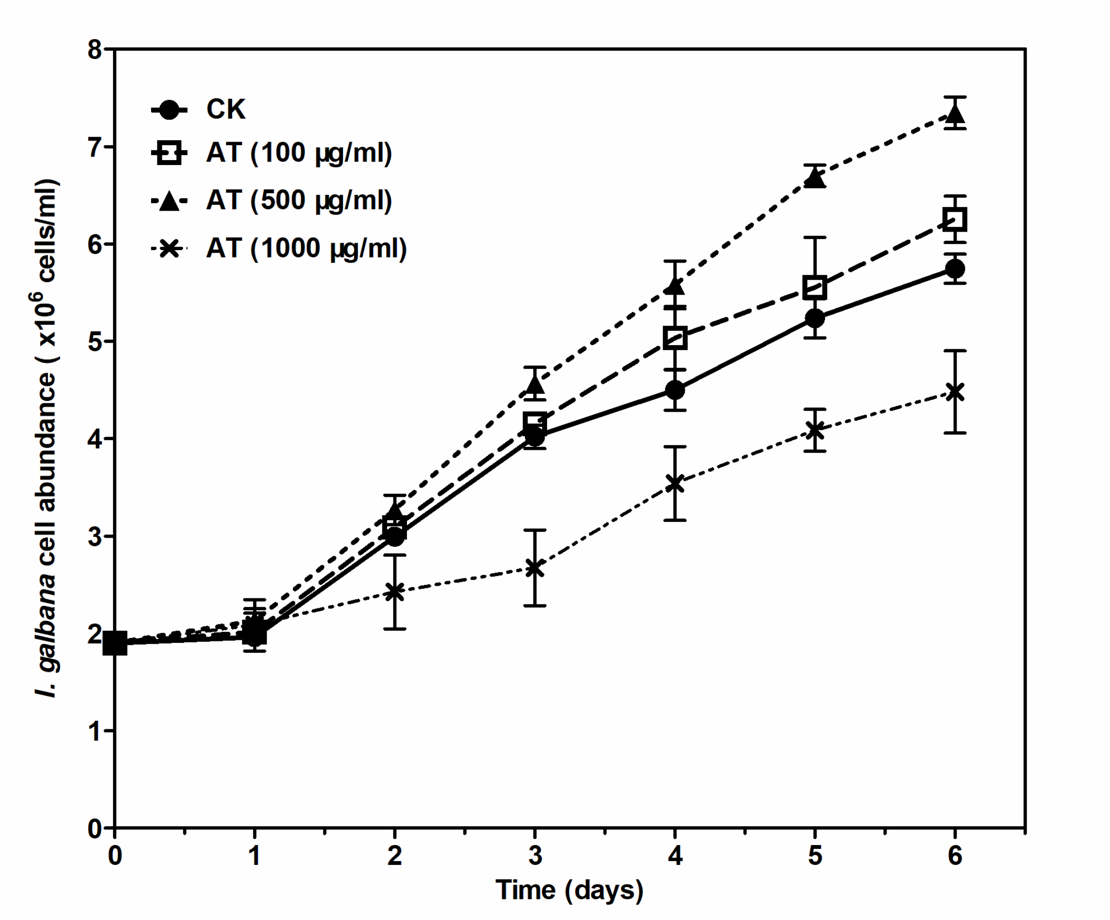


**Figure S4. Growth of *I. galbana* in the antibiotic-treated (AT) and untreated culture (CK) expressed as cell concentration.** Three concentration of ampicillin (100μg/ml, 500μg/ml and 1000 μg/ml) were used for antibiotic-treated groups. Error bars represent SE.
