# Supplementary material for: Bacterial Community Diversity and Screening of Growth-Affecting Bacteria From Isochrysis galbana Following Antibiotic Treatment
Source: Front Microbiol. 2019 May 7;10:994. doi: 10.3389/fmicb.2019.00994 (PMC6513876; doi:10.3389/fmicb.2019.00994)
Supplement: Supplementary file 5 [file Data_Sheet_3.PDF]

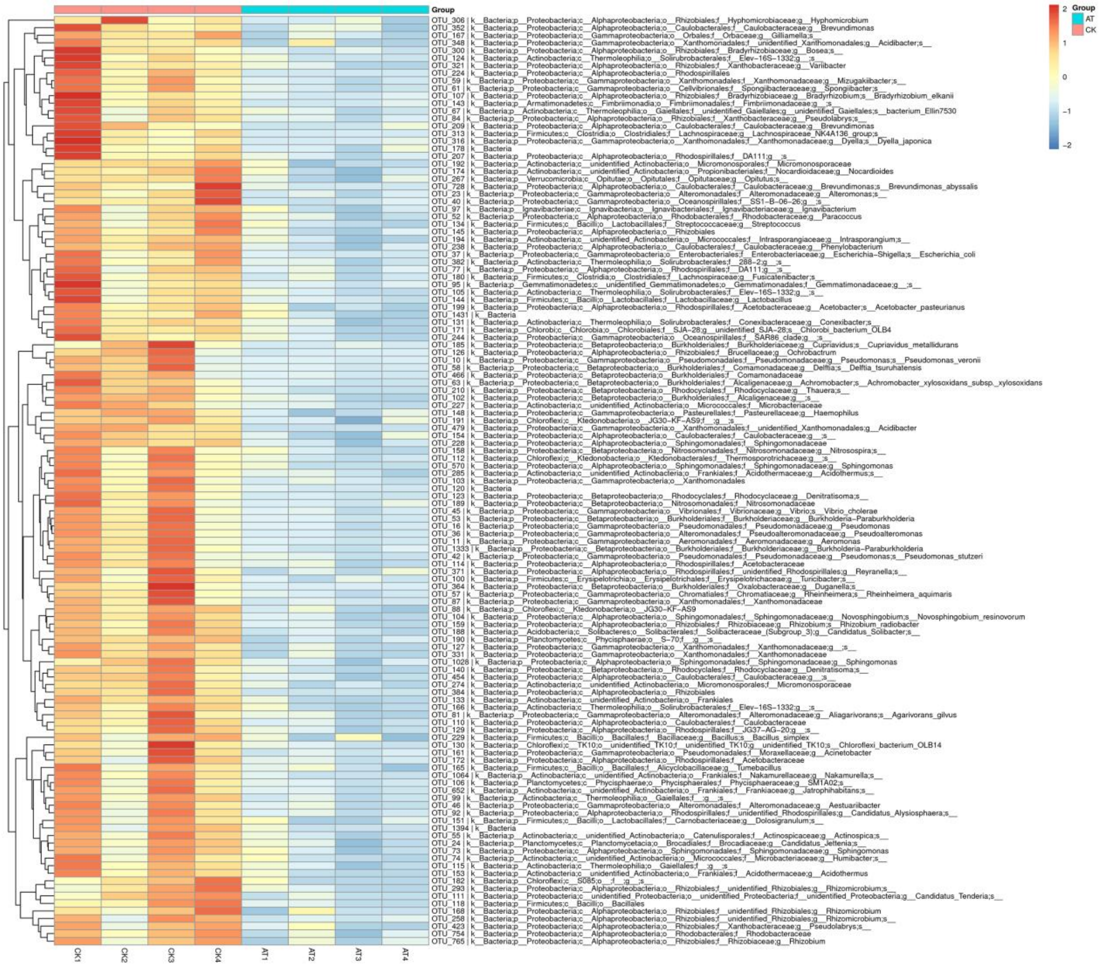

Figure S3. Heat maps showed the relative abundances of the significantly changed bacteria at the 97% OTU level based on MetaStat method. Origin of microbiota is indicated either as AT (green) or CK (red) samples. Relative abundances from high to low of bacteria were indicated with colour from red to blue.
